# Supplementary material for: The miR-19a/Cylindromatosis Axis Regulates Pituitary Adenoma Bone Invasion by Promoting Osteoclast Differentiation
Source: Cancers (Basel). 2024 Jan 11;16(2):302. doi: 10.3390/cancers16020302 (PMC10813535; doi:10.3390/cancers16020302)
Supplement: Supplementary file 1 [file cancers-16-00302-s001.zip › Supplementary table1.pdf]

Supplementary table 1. Clinical information for specimens.

Name	Patient ID	Gender	Age	Operation Date	Diagnostics	Knosp Grade	Bone Invasion
XXX	XXX	Male	46	2022/11/14	GH-PA	IIIB	Bone Invasive
XXX	XXX	Male	52	2022/11/25	GH-PA	IV	Bone Invasive
XXX	XXX	Male	41	2022/12/3	GH-PA	IV	Bone Invasive
XXX	XXX	Female	45	2022/12/24	GH-PA	IV	Bone Invasive
XXX	XXX	Male	67	2022/12/28	GH-PA	IV	Bone Invasive
XXX	XXX	Male	34	2023/1/24	GH-PA	IIIB	Bone Invasive
XXX	XXX	Male	51	2023/1/27	GH-PA	IV	Bone Invasive
XXX	XXX	Female	57	2023/2/1	GH-PA	IV	Bone Invasive
XXX	XXX	Male	49	2023/2/11	GH-PA	IV	Bone Invasive
XXX	XXX	Male	55	2023/2/20	GH-PA	IIIB	Bone Invasive
XXX	XXX	Male	60	2022/11/16	GH-PA	IV	non-Bone Invasive
XXX	XXX	Male	49	2022/12/5	GH-PA	IIIB	non-Bone Invasive
XXX	XXX	Female	57	2022/12/14	GH-PA	IV	non-Bone Invasive
XXX	XXX	Female	37	2023/1/3	GH-PA	IIIA	non-Bone Invasive
XXX	XXX	Male	49	2023/1/5	GH-PA	IIIA	non-Bone Invasive
XXX	XXX	Male	41	2023/1/17	GH-PA	IV	non-Bone Invasive
XXX	XXX	Male	47	2023/1/20	GH-PA	IIIA	non-Bone Invasive
XXX	XXX	Male	58	2023/2/2	GH-PA	IV	non-Bone Invasive
XXX	XXX	Female	53	2023/2/11	GH-PA	II	non-Bone Invasive
XXX	XXX	Male	59	2023/2/21	GH-PA	IV	non-Bone Invasive
