# Supplementary figures and images for: The miR-19a/Cylindromatosis Axis Regulates Pituitary Adenoma Bone Invasion by Promoting Osteoclast Differentiation
Source: Cancers (Basel). 2024 Jan 11;16(2):302. doi: 10.3390/cancers16020302 (PMC10813535; doi:10.3390/cancers16020302)

Figure2 F

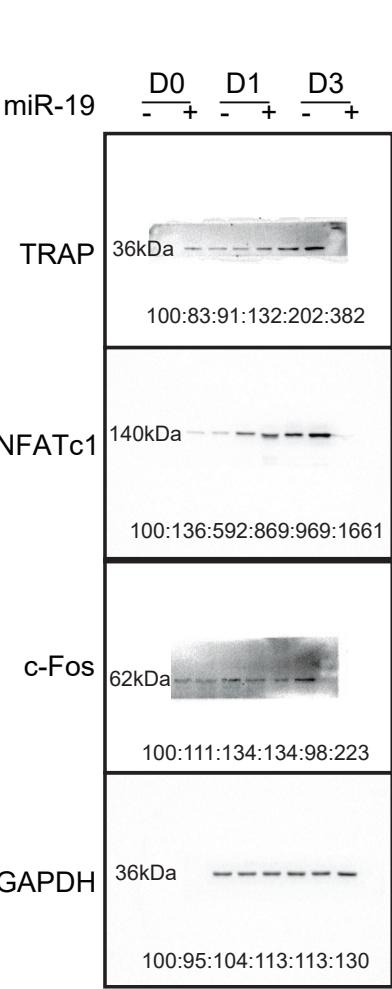

Figure3 C

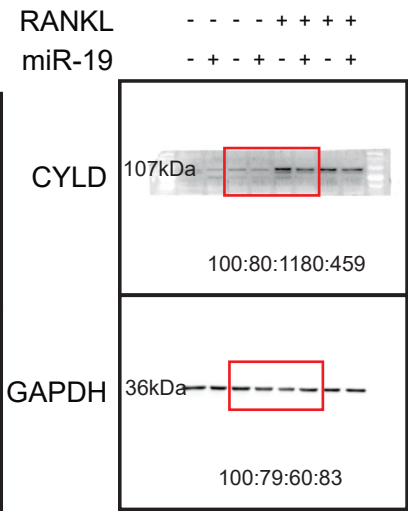

Figure3 F

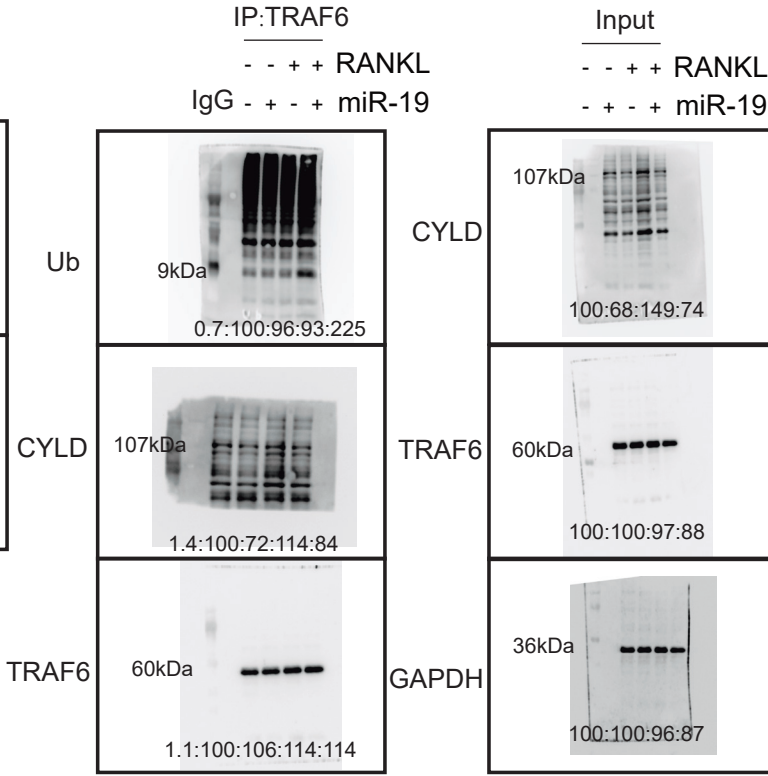

Figure5 B

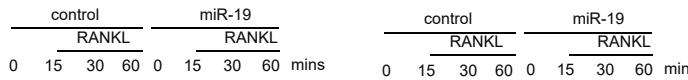

Figure4 A

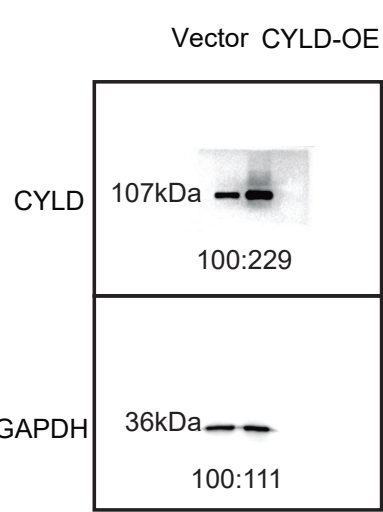

Figure5 A

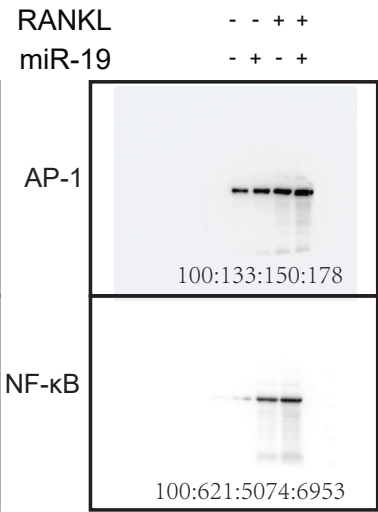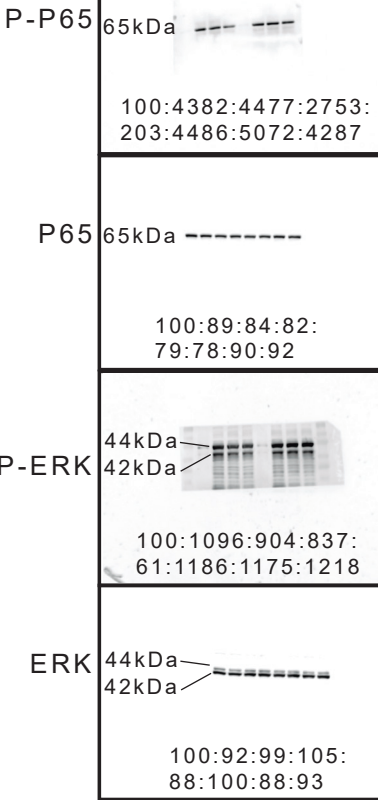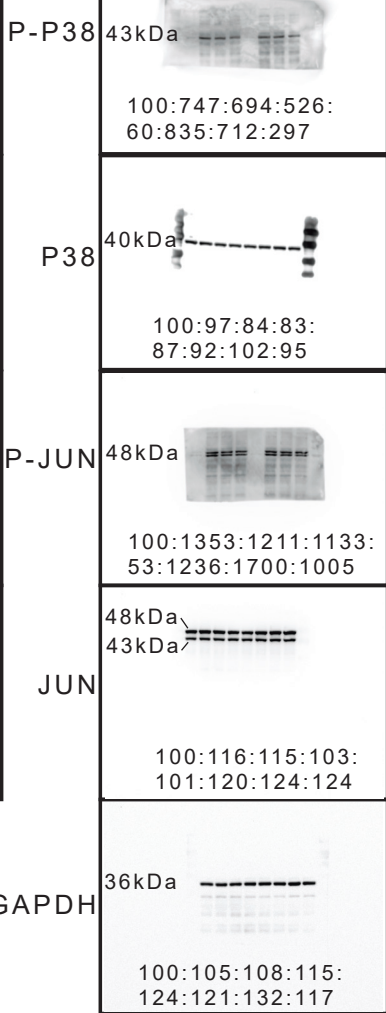

Supplement: Supplementary file 1 [file cancers-16-00302-s001.zip › Figure S1.pdf]
